# Prevalence of Drugs of Abuse and Cognitive Enhancer Consumption Monitored in Grab Samples and Composite Wastewater via Orbitrap Mass Spectrometry Analysis

**DOI:** 10.3390/molecules29163870

**Published:** 2024-08-15

**Authors:** Fabian Frankenfeld, Lea Wagmann, Cathy M. Jacobs, Markus R. Meyer

**Affiliations:** Department of Experimental and Clinical Toxicology, Institute of Experimental and Clinical Pharmacology and Toxicology, Center for Molecular Signaling (PZMS), Saarland University, 66421 Homburg, Germany

**Keywords:** wastewater-based epidemiology, wastewater surveillance, drugs of abuse, cognitive enhancers, LC-HRMS/MS

## Abstract

Wastewater (WW)-based epidemiology is an approach for the objective surveillance of the consumption of (illicit) drugs in populations. The aims of this study were to monitor drugs of abuse, cognitive enhancers, and their metabolites as biomarkers in influent WW. Data obtained from different sampling points and mean daily loads were compared with previously published data. The prevalence of analytes was monitored in WW grab samples collected monthly over 22 months at two sampling points and 24 h composite WW samples collected over 2 weeks at a WW treatment plant in the same city. Quantification was performed using a previously validated and published method based on solid-phase extraction followed by liquid chromatography coupled with high-resolution tandem mass spectrometry. Grab samples allowed for frequent detection of ritalinic acid and sporadic detection of drugs of abuse. The daily mean loads calculated for 24 h WW composite samples were in accordance with data published in an international study. Furthermore, loads of amphetamine and methamphetamine increased compared with those observed in a previously published study from 2014. This study showed frequent quantification of ritalinic acid in the grab samples, while drugs of abuse were commonly quantified in the composite WW samples. Daily mean loads were in accordance with trends reported for Germany.

## 1. Introduction

The availability and prevalence of illicit drugs remains high. The drug-use report of the European Monitoring Centre for Drugs and Drug Addiction (EMCDDA) reported that approximately 83 million Europeans aged between 15 and 64 have used illicit drugs in their life [1,2]. Amongst the most used drugs of abuse (DOA) in the European union are stimulants such as amphetamine (AMPH), 3,4-methylendioxymethamphetamine (MDMA), and cocaine (COC). In addition to DOA, prescription drugs such as methylphenidate (MPH) are abused as cognitive enhancers for stress release or performance enhancement [3]. Serious health risks are associated with acute and chronic abuse [4]. Hence, surveillance of consumption patterns and trends is of importance. Wastewater (WW)-based epidemiology is an approach to monitoring drug consumption in a population, allowing monitoring of spatial, short-, and long-term trends [5,6]. Through this approach, intake of (illicit) drugs can be monitored after their excretion into WW by detecting parent compounds and/or metabolites as biomarkers. Different sampling techniques were described in the literature; they included composite sampling, which are typically average samples collected over 24 h, or grab samples only showing the load at one single time point. Additionally, sampling over consecutive days is advised to obtain representative results [7]. In 2011, the first Europe-wide WW investigation was conducted by the sewage analysis CORe group—Europe (SCORE), providing data about illicit drug consumption in 19 European cities. Since then, annual reports have been published, including data from several German cities [8]. The aim of this study was the local monitoring of concentrations and loads of amphetamine, benzoylecgonine (BZE), cocaethylene (CE), cocaine, MDMA, methamphetamine (METH), methylphenidate, and ritalinic acid (RA) in influent WW collected as grab or 24 h composite samples. Data obtained at the different sampling points and mean daily loads calculated for 24 h composite samples should be compared with previously published data to identify drug consumption trends.

## 2. Results and Discussion

Concentrations of AMPH, BZE, and MDMA were quantified using a C_18_ column and CE, COC, METH, MPH, and RA using a HILIC column. The development, validation, and results, based on which the concentration values of the monitored compounds were determined, have already been published [9]. The current method was operated at 5 ppm mass tolerance in positive ESI mode only, as all analytes could be detected sufficiently. It should be noted that some compounds such as ethyl sulfate can only be detected sufficiently by using negative ESI. Furthermore, negative ESI is often less prone to matrix effects than positive ESI [10]. The in-sewer (WW “surviving” time) stability of all analytes was already tested and found to be sufficient [11,12,13]. For preparation of the blank samples, purified water and surface water were used and they were found to be free of analytes.

### 2.1. Wastewater Grab Samples

**Figure 1:**
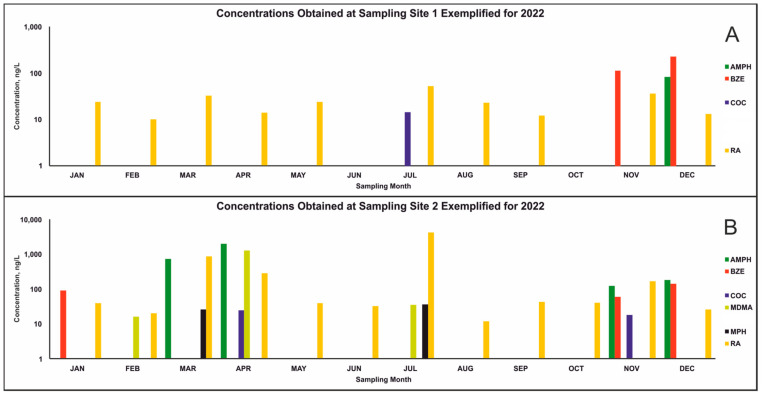
Concentrations of drugs of abuse, one cognitive enhancer, and their metabolites as biomarkers in wastewater grab samples exemplified for 2022, in logarithmic scale. (**A**) Results of sampling site one; (**B**), results of sampling site two. AMPH—amphetamine; BZE—benzoylecgonine; CE—cocaethylene; COC—cocaine; MDMA—3,4-methylenedioxymethamphetamine; METH—methamphetamine; MPH—methylphenidate; RA—ritalinic acid.

Quantification results of all (*n =* 44) grab samples are summarized in Table S1 in the Electronic Supplementary Material (ESM). Figure 1 compares the 2022 results obtained at both sites. Methylphenidate (as MPH or RA) was the most frequently detected compound (39/44 samples). MPH concentrations ranged between 11 and 34 ng/L, while its metabolite RA ranged from 10 to 3869 ng/L. Cocaine (as COC or BZE) was the second most frequently detected in a total of 23/44 samples. COC ranged between 12 and 729 ng/L and BZE between 56 and 246 ng/L. AMPH appeared in 9/44 samples with concentrations between 56 and 1814 ng/L. MDMA was detected in 8/44 samples and quantified between 15 and 1155 ng/L. CE and METH could not be detected. Quantified values below the respective lower limits of quantification (LLOQ, 30 ng/L for BZE and 10 ng/L for MDMA) were specified as < “LLOQ” in Table S1. Calculated values below the LLOQ were not considered in Figure 1 and the comparison of the sampling sites. The frequent detection of RA may be due to the predominant prescription of MPH for ADHD patients in Germany [14]. However, misuse of MPH as a cognitive enhancer was also reported [15]. Moreover, Letzel et al. reported low removal rates of RA during sewage treatment, leading to RA concentrations in the ng/L range in effluent WW [11]. Effluent WW is released into rivers and other bodies; it can also be used to treat drinking water and then discharged into the sewage system, which ultimately might influence quantification results [16]. The absence of METH in the samples is in accordance with previously published data, showing only low METH concentrations in the western part of Germany [5,6]. As grab samples do not allow for normalization of concentrations and are only representative for the time of sampling, comparison with data from other studies is not possible. Furthermore, according to Ort et al., the absence of analytes in grab samples does not prove their total absence at this sampling site, since only the time point of sampling is represented [7].

### 2.2. Twenty-Four Hour Composite Wastewater Samples

**Figure 2:**
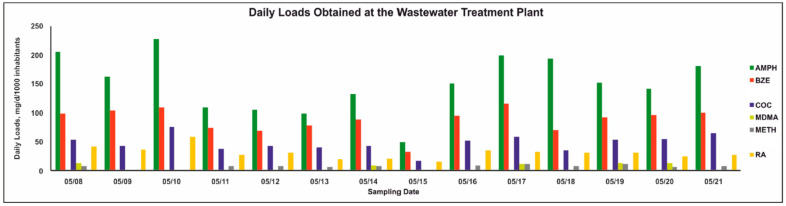
Daily loads of drugs of abuse, one cognitive enhancer, and their metabolites as biomarkers in 24 h composite wastewater samples throughout weeks 19 and 20 in May 2023. AMPH—amphetamine; BZE—benzoylecgonine; CE—cocaethylene; COC—cocaine; MDMA—3,4-methylenedioxymethamphetamine; METH—methamphetamine; MPH—methylphenidate; RA—ritalinic acid.

Daily loads (mg/day/1000 inhabitants) of all analytes, as well as daily, weekday, and weekend mean values are provided in Table S2 (ESM) and compared in Figure 2. AMPH, BZE, COC, and RA were found in all samples, while MDMA and METH were found in 5/14 and 11/14 samples, respectively. CE and MPH could not be detected. Comparison with a study by Meyer et al. showed an increase in the mean daily load of AMPH to 149 mg/day/1000 inhabitants compared with the median daily load of 46 mg/day/1000 inhabitants found in 2014 [6]. This observation is consistent with data provided by the EMCDDA and SCORE network [8]. Daily mean loads of AMPH in Saarbrucken (SB), which is also located in the federal state of Saarland, showed an even higher daily mean value of 346 mg/day/1000 inhabitants. Data from 2017 onwards showed increasing daily mean loads for AMPH in SB up to 2023, and highest AMPH loads in Europe were, amongst others, detected in German cities [8]. The daily mean load of METH was 5 mg/day/1000 inhabitants in this study, also showing an increase compared with the median published by Meyer et al. with 1.5 mg/day/1000 inhabitants [6]. These values are comparable to those obtained for SB (1.6 mg/day/1000 inhabitants), but below loads in the eastern part of Germany, e.g., Chemnitz, with 346 mg/day/1000 inhabitants [8]. MDMA (4 mg/day/1000 inhabitants) was also below the load obtained in SB (26 mg/day/1000 inhabitants) and below the lowest German daily mean (Munich, 7 mg/day/1000 inhabitants). Zuccato et al. proposed correction factors for AMPH and related substances and for BZE/COC, considering their excretion in humans [17]. Using these factors, the corrected daily mean load of AMPH would be 492 mg/day/1000 inhabitants, METH at 11.5 mg/day/1000 inhabitants, and MDMA at 6 mg/day/1000 inhabitants. Adjustment of METH and MDMA loads does not lead to a relevant change in comparison with loads provided by the EMCDDA, but the adjusted load of AMPH is higher compared with SB in the EMCDDA study [8]. Furthermore, a potential overestimation of AMPH due to METH metabolism, resulting in up to 7% AMPH, was discussed by van Nuijs et al. [18]. Due to low METH loads and the mentioned distribution of AMPH and METH in Germany, an overestimation should not be of relevance. The daily mean load of COC was below the values provided for SB in the EMCDDA report, with 46 mg/day/1000 inhabitants in Homburg compared with 245 mg/day/1000 inhabitants in SB and below the lowest daily mean in Germany (Chemnitz, 57 mg/day/1000 inhabitants) [8]. Considering the daily mean load of BZE (86 mg/day/1000 inhabitants), the ratio of BZE to COC was 0.53. A study by Castiglioni et al. showed similar ratios of both analytes in WW samples in Italy and the USA [19]. Using the correction factor for BZE to estimate COC loads described by Zuccato et al., the mean daily load of COC would be at 200 mg/day/1000 inhabitants [17]. This corrected value is higher compared with the aforementioned load of COC using the quantified COC concentrations and closer to the load described for SB. Comparison of RA daily means (29 mg/day/1000 inhabitants) with a study by Letzel et al. showed comparable results for influent WW obtained at a WWTP in Southern Bavaria serving 26,000 inhabitants (23.7 mg/day/1000 inhabitants) [11]. During the reported sampling period, weekday means of AMPH, BZE, and COC were slightly higher compared with weekend means. This might be explained by the fact that there were no festivals on either weekend or that the only holiday was on a weekday (Thursday, 05/18/23).

### 2.3. Comparison of Grab Samples and 24 h Composite Samples

Comparison of qualitative results obtained in both grab and 24 h composite samples revealed that AMPH, BZE, COC, and RA were continuously detectable in all 24 h composite samples. Grab samples allowed only sparse detection of most analytes, except RA, which could be detected in almost all samples (39/44). As already discussed in Section 2.1, this might be due to low removal rates [11]. METH was not detected using grab samples but was quantified in 24 h composite samples. This could be explained by only seldom and occasional consumption of METH within the monitored community. This would mean that METH was not detected in grab samples at this sampling time but may have been detected at another point in time. As CE was neither detected in grab nor 24 h composite samples, it is possible that no formation of CE took place during the monitored sampling time.

## 3. Materials and Methods

### 3.1. Reagents and Materials

Analytes and their deuterated analogues, used as internal standards (IS), were obtained from LGC (Wesel, Germany). All other chemicals were purchased from VWR (Darmstadt, Germany). Water was purified with a Millipore (Merck, Darmstadt, Germany) filtration unit, which purifies water to a resistance of 18.2 Ω × cm. Stock solutions, calibrator, IS mix, and quality control working solutions were prepared as described elsewhere [9].

### 3.2. Sample Preparation

Sample preparation via solid-phase extraction was performed according to a previously published procedure [9]: 10 mL WW samples were fortified with IS mix solution (final concentration 50 ng/L, except BZE-d_3_ 150 ng/L). Particles were removed using Phenex-PTFE 25-mm syringe filters 0.2 mm (Phenomenex, Aschaffenburg, Germany) and SPE was performed using Isolute 200 mg/10 mL (3 mL XL) HCX cartridges (Biotage, Uppsala, Sweden). The cartridges were primed with 1 mL methanol (MeOH) and 1 mL purified water, then cartridges were loaded with 10 mL of WW followed by a washing step using purified water, 0.1 M hydrochloric acid, and MeOH (1 mL each). Compounds were eluted using 1.25 mL of a MeOH/NH_3_ mixture (35%, 98/2, *v*/*v*). Eluates were partitioned into two equal aliquots and evaporated to dryness under a gentle stream of nitrogen at 40 °C. Residues were reconstituted using a mixture of water and formic acid (50 µL, 99/1, *v*/*v*, RP C_18_ samples) or a mixture of acetonitrile and formic acid (50 µL, 99/1, *v*/*v*, HILIC samples). Calibration and quality control samples were prepared accordingly; final concentrations of calibrators and quality controls are given in Table S3.

### 3.3. Instrumental Settings

Samples were analyzed according to a previously published procedure with minor changes [9], using a Thermo Fisher Scientific (TF, Dreieich, Germany) Dionex UltiMate 3000 consisting of a degasser, a quaternary pump, a DL W2 wash system, and an HCT PAL autosampler (CTC Analytics AG, Zwinger, Switzerland). The system was coupled with a TF Q Exactive orbitrap mass spectrometer equipped with a heated electrospray ionization II source (HESI-II). Calibration was performed prior to analysis according to the manufacturer’s recommendations using external mass calibration. The final conditions of the LC-system using the C_18_ column were as follows: Waters AQUITY UPLC BEH C18 column (100 mm × 2.1 mm, 1.7 µm; Milford, MA, USA); gradient elution was performed with 2 mM ammonium formate solution containing 0.1% (*v*/*v*) formic acid (eluent A) and 2 mM ammonium formate solution in ACN/MeOH (50/50, *v*/*v*) containing 0.1% (*v*/*v*) formic acid and 1% (*v*/*v*) water (eluent B). The flow rate was 0.500 mL/min with the following gradient settings: 0–1 min 85% A, 1–3 min to 40% A, 3–6 min to 30% A, 6–8 min 1% A, 8–9.2 min hold 1% A, 9.2–9.21 min to 85% A, 9.21–10 min hold to 85% A.

The final conditions of the LC system with the HILIC column were as follows: Merck SeQuant ZIC-cHILIC column (150 × 2.1 mm, 3 µm; Merck, Darmstadt, Germany); gradient elution with 200 mM aqueous ammonium acetate solution (eluent C) and ACN containing formic acid 0.1% (*v*/*v*) (eluent D). The flow rate was at 0.5 mL/min with the following gradient settings: 0–1 min hold to 1% C, 1–1.8 min to 10% C, 1.8–9 min hold to 10% C, 9–9.5 min to 60% C, 9.5–10.5 min hold to 60% C, 10.5–10.6 min to 1% C, and 10.6–15 min hold to 1% C. Chromatography on both columns was performed at 40 °C.

The final HESI-II source and MS conditions were as follows: ionization mode, positive; sheath gas, 60 arbitrary units (AU); auxiliary gas flow rate, 10 AU; spray voltage, 4.00 kV; auxiliary gas heater temperature, 320 °C; ion capillary temperature, 320 °C; and S-lens RF level, 60.0. Mass spectrometry experiments after C_18_ column separation were performed using parallel reaction monitoring (PRM) in positive mode with a scheduled inclusion list containing the precursor masses of interest, adjusted normalized collision energies (NCE). The settings for PRM experiments were as follows: resolution, 17,500; automatic gain control target, 2 × 10^5^; maximum injection time, 250 ms; isolation window, 1 *m*/*z*; high-energy collisional dissociation with NCE 30, 40 eV. Mass spectrometry experiments after HILIC column separation were performed using PRM in positive mode as described for the C_18_ column. TF Xcalibur Qual Browser version 4.1.31.9 was used for data handling. Masses of the precursor ions (*m*/*z*) used for the inclusion list, polarity, and adjusted NCE were previously described [9] and are also given in Table S4. The MS2 spectra of all analytes are shown in Figure S1.

### 3.4. Sampling

#### 3.4.1. Wastewater Grab Samples

WW grab samples (*n* = 44 in total) were obtained between June 2021 and March 2023 at two different sewers (sampling sites one and two) in the city of Homburg, Germany. Samples (80 mL total sample volume each) were acidified with acetic acid (0.1%, *v*/*v*) and stored at −22 °C until sample preparation. If quantification results were above the calibration range, samples were diluted 1:10 with blank surface water and then analyzed according to Section 3.2 and Section 3.3.

#### 3.4.2. Twenty-Four Hour Composite Wastewater Samples

In total, fourteen 24 h composite influent WW samples were obtained using an Endres + Hauser stationary water samples type asp-station 2000. The sampling point was located immediately after the grit chamber, and the total sample volume was 600 mL and consisted of twelve 50 mL samples collected every two hours. The sample interval was from 8 am to 8 am, throughout weeks 19 and 20 in May 2023 at the WW treatment plant (WWTP) in Homburg, Germany. Finally, 80 mL of each 24 h composite sample was acidified and stored as described in Section 3.4.1.

## 4. Conclusions

In the presented study, we analyzed concentrations of DOA, one cognitive enhancer, and their metabolites in influent WW samples by comparing grab samples from two different sampling sites and 24 h composite samples. RA, AMPH, BZE, and COC were the most frequently quantified analytes in grab samples. Compared with a previous study [6], 24 h composite samples revealed increasing loads of AMPH and METH at the same WWTP, which is in line with data published for other cities in Germany [8]. However, METH loads were lower compared with cities in the eastern part of Germany, due to the European AMPH/METH borders location Germany [6]. Furthermore, loads of COC and MDMA were also in accordance with values obtained in German cities [8]. Comparison of grab samples and 24 h composite samples indicated that composite samples provided more consistent detection and quantification results of analytes. These findings show the importance of the sampling strategies used in the context of WW-based epidemiology. The rise of DOA loads observed in Homburg in this study also underlines the importance of continuous WW surveillance to observe trends even in smaller communities.

## Data Availability

The data presented in this study are available on request from the corresponding author.

## Supplementary Materials

The following supporting information can be downloaded at: https://www.mdpi.com/article/10.3390/molecules29163870/s1, Figure S1: MS2 spectra of all analytes; Table S1: Concentrations of drugs of abuse, one cognitive enhancer, and their metabolites as biomarkers in wastewater grab samples between June 2021 and March 2023.; Table S2: Daily loads of drugs of abuse, one cognitive enhancer, and their metabolites as biomarkers as well as daily, weekday, and weekend mean values in 24 h composite wastewater samples throughout weeks 19 and 20 in May 2023.; Table S3: Final concentrations of the calibrators (Cal) and quality control (QC) samples, lower limit of quantification (LLOQ) and upper limit of quantification (ULOQ) in surface water; Table S4: Analytes and internal standards included in the method validation, the m/z of their precursor and quantifier ions.
