# Supplementary material for: Prevalence of Drugs of Abuse and Cognitive Enhancer Consumption Monitored in Grab Samples and Composite Wastewater via Orbitrap Mass Spectrometry Analysis
Source: Molecules. 2024 Aug 15;29(16):3870. doi: 10.3390/molecules29163870 (PMC11357585; doi:10.3390/molecules29163870)
Supplement: Supplementary file 1 [file molecules-29-03870-s001.zip › molecules-3122647-supplementary.pdf]

Prevalence of Drugs of Abuse and Cognitive Enhancer Consumption Monitored in Grab and Composite Wastewater by Means of Orbitrap Mass Spectrometry Analysis

Fabian Frankenfeld, Lea Wagmann, Cathy M. Jacobs, Markus R. Meyer*

***Corresponding author: Prof. Dr. Markus R. Meyer**, Department of Experimental and Clinical Toxicology and Pharmacology, Institute of Experimental and Clinical Pharmacology and Toxicology, Center for Molecular Signaling (PZMS), Saarland University, Kirrberger Str., Building 46, 66421 Homburg, Germany, Phone: +49-6841-16-26430, Fax: +49-6841-16-26431, E-mail: m.r.meyer@mx.uni-saarland.de

Fabian Frankenfeld, Dr. Lea Wagmann, Cathy M. Jacobs: Department of Experimental and Clinical Toxicology and Pharmacology, Institute of Experimental and Clinical Pharmacology and Toxicology, Center for Molecular Signaling (PZMS), Saarland University, Homburg

Table S1. Concentrations of drugs of abuse, one cognitive enhancer, and their metabolites as biomarkers in wastewater grab samples between June 2021 and March 2023. AMPH, amphetamine; BZE, benzoylecgonine; CE, cocaethylene; COC, cocaine; MDMA, 3,4-methylenedioxymethamphetamine; METH, methamphetamine; MPH, methylphenidate; n. d., not detected; RA, ritalinic acid; #, back calculated concentration obtained after 1:10 dilution of samples; *, concentration remaining above the calibration range after 1:10 dilution; concentrations below the respective lower limit of quantification (LLOQ) are given as "< LLOQ"

Sampling month	Sampling site	Analyte concentration, ng/L							
		AMPH	BZE	CE	COC	MDMA	METH	MPH	RA
06/21	one	n. d.	n. d.	n. d.	n. d.	n. d.	n. d.	n. d.	n. d.
	two	n. d.	n. d.	n. d.	n. d.	n. d.	n. d.	n. d.	18
07/21	one	n. d.	< 30	n. d.	n. d.	n. d.	n. d.	n. d.	85
	two	n. d.	n. d.	n. d.	n. d.	n. d.	n. d.	n. d.	n. d.
08/21	one	n. d.	n. d.	n. d.	n. d.	n. d.	n. d.	n. d.	16
	two	n. d.	< 30	n. d.	n. d.	n. d.	n. d.	n. d.	17
09/21	one	n. d.	n. d.	n. d.	n. d.	n. d.	n. d.	11	586#
	two	720#	246	n. d.	729#	n. d.	n. d.	n. d.	55
10/21	one	n. d.	n. d.	n. d.	n. d.	n. d.	n. d.	n. d.	36
	two	1,385#*	n. d.	n. d.	22	n. d.	n. d.	n. d.	366#
11/21	one	n. d.	94	n. d.	12	16	n. d.	n. d.	23
	two	n. d.	241	n. d.	15	n. d.	n. d.	n. d.	483#
12/21	one	n. d.	n. d.	n. d.	n. d.	n. d.	n. d.	n. d.	25
	two	88	< 30	n. d.	17	n. d.	n. d.	n. d.	17
01/22	one	n. d.	< 30	n. d.	n. d.	n. d.	n. d.	n. d.	24
	two	n. d.	83	n. d.	n. d.	n. d.	n. d.	n. d.	36
02/22	one	n. d.	< 30	n. d.	n. d.	n. d.	n. d.	n. d.	10
	two	n. d.	< 30	n. d.	n. d.	15	n. d.	n. d.	19
03/22	one	n. d.	< 30	n. d.	n. d.	n. d.	n. d.	n. d.	33
	two	665#	< 30	n. d.	n. d.	n. d.	n. d.	24	783#
04/22	one	n. d.	< 30	n. d.	n. d.	n. d.	n. d.	n. d.	14
	two	1,814#*	< 30	n. d.	23	1155#*	n. d.	n. d.	261#

Table S1. continued

Sampling month	Sampling site	Analyte concentration, ng/L							
		AMPH	BZE	CE	COC	MDMA	METH	MPH	RA
05/22	one	n. d.	n. d.	n. d.	n. d.	n. d.	n. d.	n. d.	24
	two	n. d.	n. d.	n. d.	n. d.	n. d.	n. d.	n. d.	36
06/22	one	n. d.	n. d.	n. d.	n. d.	< 10	n. d.	n. d.	n. d.
	two	n. d.	< 30	n. d.	n. d.	n. d.	n. d.	n. d.	30
07/22	one	n. d.	n. d.	n. d.	15	n. d.	n. d.	n. d.	53
	two	n. d.	n. d.	n. d.	n. d.	33	n. d.	34	3869[#]*
08/22	one	n. d.	n. d.	n. d.	n. d.	< 10	n. d.	n. d.	23
	two	n. d.	n. d.	n. d.	n. d.	n. d.	n. d.	n. d.	11
09/22	one	n. d.	n. d.	n. d.	n. d.	n. d.	n. d.	n. d.	12
	two	n. d.	n. d.	n. d.	n. d.	n. d.	n. d.	n. d.	40
10/22	one	n. d.	n. d.	n. d.	n. d.	n. d.	n. d.	n. d.	n. d.
	two	n. d.	n. d.	n. d.	n. d.	n. d.	n. d.	n. d.	38
11/22	one	n. d.	120	n. d.	n. d.	n. d.	n. d.	n. d.	36
	two	113[#]	56	n. d.	17	n. d.	n. d.	n. d.	155[#]
12/22	one	87	239	n. d.	n. d.	n. d.	n. d.	n. d.	13
	two	168[#]	131	n. d.	n. d.	n. d.	n. d.	n. d.	24
01/23	one	n. d.	n. d.	n. d.	n. d.	n. d.	n. d.	n. d.	n. d.
	two	n. d.	n. d.	n. d.	n. d.	n. d.	n. d.	n. d.	31
02/23	one	n. d.	128	n. d.	n. d.	n. d.	n. d.	n. d.	18
	two	n. d.	n. d.	n. d.	n. d.	44	n. d.	n. d.	21
03/23	one	n. d.	n. d.	n. d.	n. d.	n. d.	n. d.	n. d.	769[#]
	two	56	n. d.	n. d.	12	< 10	n. d.	n. d.	28

Table S2. Daily loads of drugs of abuse, one cognitive enhancer, and their metabolites as biomarkers as well as daily, weekday and weekend mean values in 24-h composite wastewater samples throughout weeks 19 and 20 in May 2023. AMPH, amphetamine; BZE, benzoylecgonine; CE, cocaethylene; COC, cocaine; MDMA, 3,4-methylenedioxymethamphetamine; METH, methamphetamine; MPH, methylphenidate; n. d., not detected; RA, ritalinic acid, -; not applicable.

Sampling date	Day	Daily loads, mg/day/1000 inhabitants							
		AMPH	BZE	CE	COC	MDMA	METH	MPH	RA
05/08/23	Monday	203	98	n. d.	51	12	6	n. d.	40
05/09/23	Tuesday	161	102	n. d.	42	n. d.	n. d.	n. d.	35
05/10/23	Wednesday	226	108	n. d.	73	n. d.	n. d.	n. d.	57
05/11/23	Thursday	107	72	n. d.	36	n. d.	6	n. d.	25
05/12/23	Friday	103	67	n. d.	41	n. d.	6	n. d.	30
05/13/23	Saturday	97	76	n. d.	39	n. d.	5	n. d.	18
05/14/23	Sunday	131	87	n. d.	41	8	6	n. d.	19
05/15/23	Monday	47	31	n. d.	15	n. d.	n. d.	n. d.	14
05/16/23	Tuesday	149	93	n. d.	51	n. d.	8	n. d.	34
05/17/23	Wednesday	196	114	n. d.	56	11	10	n. d.	31
05/18/23	Thursday	191	69	n. d.	33	n. d.	6	n. d.	30
05/19/23	Friday	150	91	n. d.	51	12	10	n. d.	30
05/20/23	Saturday	140	95	n. d.	53	11	5	n. d.	24
05/21/23	Sunday	179	99	n. d.	63	n. d.	7	n. d.	25
Daily mean	-	149	86	-	46	4	5	-	29
Weekday mean	-	153	84	-	45	3	5	-	33
Weekend mean	-	137	89	-	49	5	6	-	21

Table S3

Final concentrations of the calibrators (Cal) and quality control (QC) samples, lower limit of quantification (LLOQ) and upper limit of quantification (ULOQ) in surface water, concentrations are given in ng/L; mid: medium; AMPH: amphetamine; BZE: benzoylecgonine; CE: cocaethylene; COC: cocaine; MDMA: 3,4-methylenedioxymethamphetamine; METH: methamphetamine; MPH: methylphenidate; RA: ritalinic acid

Analyte	Cal 1 (LLOQ)	Cal 2	Cal 3	Cal 4	Cal 5	Cal 6 (ULOQ)	QC LLOQ	QC low	QC mid	QC high
AMPH	10	20	40	60	80	100	10	30	50	90
BZE	30	60	120	180	240	300	30	90	150	270
CE	10	20	40	60	80	100	10	30	50	90
COC	10	20	40	60	80	100	10	30	50	90
MDMA	10	20	40	60	80	100	10	30	50	90
METH	10	20	40	60	80	100	10	30	50	90
MPH	10	20	40	60	80	100	10	30	50	90
RA	10	20	40	60	80	100	10	30	50	90

Table S4

Analytes and internal standards included in the method validation, the *m/z* of their precursor and quantifier ions, HILIC: hydrophilic interaction liquid chromatography; limit of identification (LOI) and retention times (RT) on both columns; AMPH: amphetamine; BZE: benzoylecgonine; CE: cocaethylene; COC: cocaine; MDMA: 3,4-methylenedioxymethamphetamine; METH: methamphetamine; MPH: methylphenidate; n. t.: not tested; RA: ritalinic acid

Analyte	Precursor ion, *m*	Quantifier ion, *m/*:	LOI, [ng/L]	RT (HILIC, min	RT (C_{18}, min	NCE [eV
AMPH	136.1121	91.0542	10	5.36	1.75	40
AMPH-d_5	141.1435	93.0672	n. t.	5.36	1.75	40
BZE	290.1387	168.1019	1	2.26	3.25	30
BZE-d_3	293.1575	171.1205	n. t.	2.26	3.25	30
CE	318.1700	196.1332	1	0.92	4.72	30
CE-d_8	326.2202	204.1834	n. t.	0.92	4.72	30
COC	304.1543	182.1175	1	0.95	4.33	30
COC -d_3	307.1732	185.1363	n. t.	0.95	4.33	30
MDMA	194.1176	163.0753	1	4.64	2.04	40
MDMA-d_5	199.1489	165.0879	n. t.	4.64	2.04	40
METH	150.1277	91.0542	1	4.64	2.04	40
METH-d_5	155.1591	92.0608	n. t.	4.64	2.04	40
MPH	234.1489	84.0814	1	1.95	4.20	30
MPH-d_9	243.2053	93.1377	n. t.	1.95	4.20	30
RA	220.1332	84.0814	1	6.32	3.17	30
RA-d_{10}	230.1960	93.1377	n. t.	6.32	3.17	30

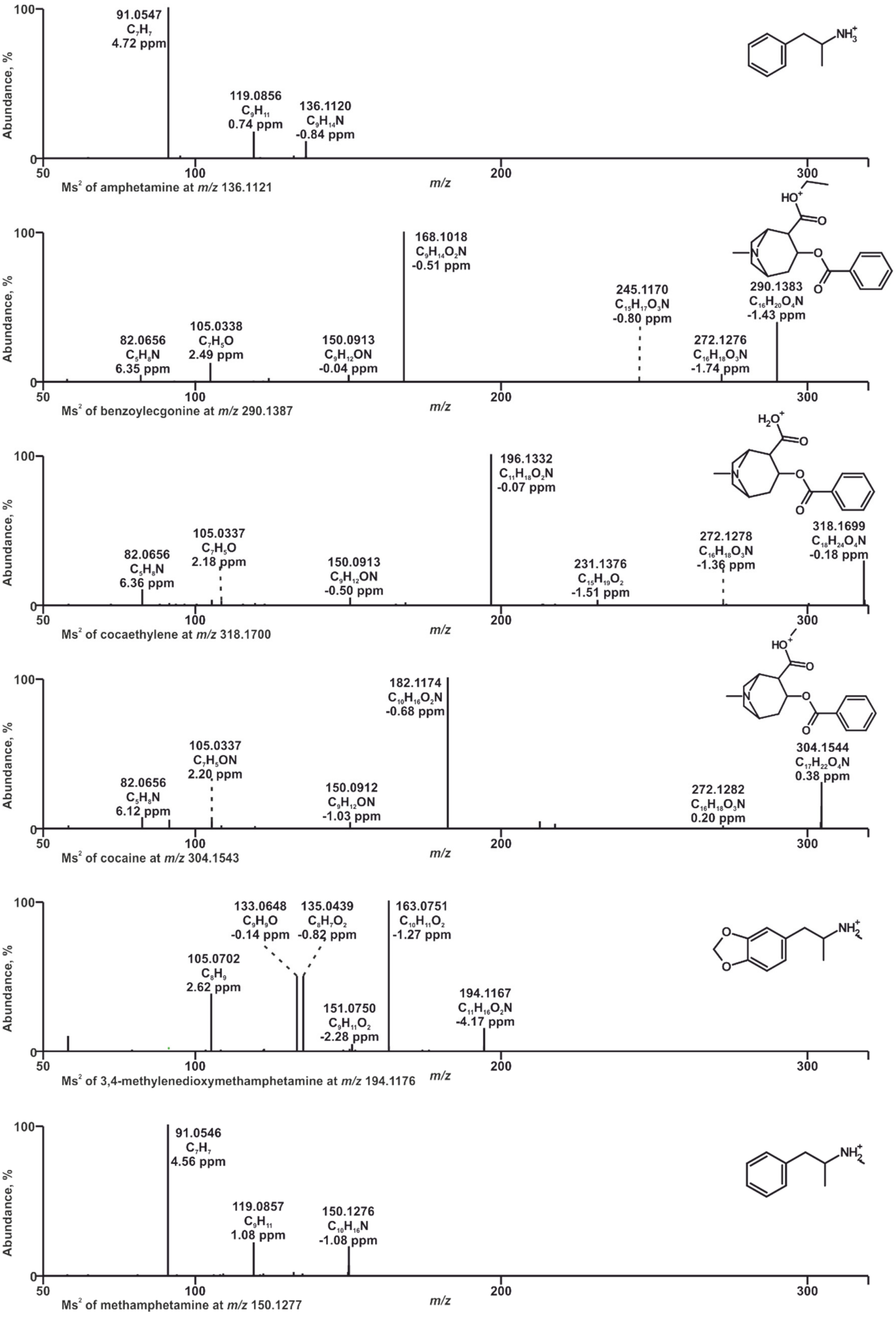

Figure S1. $HRMS^2$ spectra of the compounds of interest.

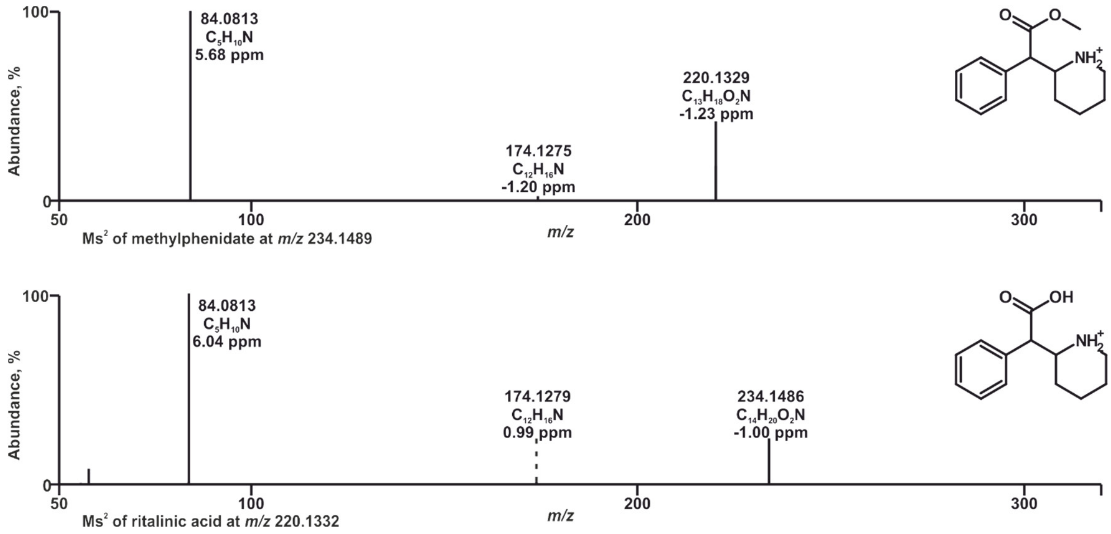

Figure S2. Continued.
